# Cerebrospinal fluid levels of IL-6 are decreased and correlate with cognitive status in DLB patients

**DOI:** 10.1186/s13195-015-0145-y

**Published:** 2015-10-05

**Authors:** Malin Wennström, Sara Hall, Katarina Nägga, Elisabet Londos, Lennart Minthon, Oskar Hansson

**Affiliations:** Clinical Memory Research Unit, Department of Clinical Sciences Malmö, Faculty of Medicine, Lund University, Wallenberg Laboratory, floor 2, Inga-Marie Nilssons gata 53, 205 02 Malmö, Sweden

## Abstract

**Introduction:**

Inflammatory processes have previously been shown to influence cognition and progression of dementia. An involvement of interleukin (IL)-6 has in particular been suggested as altered levels of IL-6 in cerebrospinal fluid (CSF) have been found in patients with Alzheimer’s disease (AD). Also, an association between cognitive decline and levels of IL-6 in CSF have been reported. The aim of the present study was to investigate whether patients clinically diagnosed with dementia with Lewy bodies (DLB) display altered CSF IL-6 levels in comparison with patients with AD and control subjects without dementia and whether the IL-6 levels are correlated with cognitive status and biomarkers for AD and synucleinopathy.

**Methods:**

To analyse CSF of patients with AD (n = 45), patients with DLB (n = 29) and control subjects without dementia (n = 36), we used immunoassays to measure levels of IL-6 (multiplex electrochemiluminescence); AD markers phosphorylated tau, total tau and amyloid-β_1–42_ (enzyme-linked immunosorbent assay [ELISA]); and α-synuclein (ELISA). Cognitive status was evaluated using the Mini Mental State Examination (MMSE).

**Results:**

Our analysis showed significantly lower levels of IL-6 in CSF from patients with DLB than in CSF from patients with AD and control subjects without dementia. The IL-6 levels were also negatively correlated with MMSE and positively correlated with α-synuclein CSF levels.

**Conclusions:**

Our findings support previous studies by demonstrating a link between inflammatory processes and dementia progression and further strengthen the hypothesis that IL-6 is involved in dementia pathology and cognitive decline.

## Introduction

Glial secretion of interleukin-6 (IL-6), along with other pro- and anti-inflammatory cytokines and chemokines, is drastically increased in response to infection or harmful changes in the brain. The increase in IL-6 secretion has been shown to be beneficial [1–5], but sometimes also detrimental [6–9], for the neuronal network, depending on variables such as cytokine concentration, brain region, target cell and developmental stage. The impact of IL-6 on neuronal network formation and function is noticeable in a number of in vivo rodent studies. For example, foetal exposure to IL-6 has been shown to alter rat hippocampal structure and impair spatial learning [10], and deficiency of IL-6 has been shown to improve long-term memory [11] and lipopolysaccharide-induced impairment of working memory [12]. Also, results of clinical studies point towards a relationship between IL-6 and brain function, as cognitive decline has been associated with elevated levels of IL-6 in plasma and/or serum [13–16] and cerebrospinal fluid (CSF) [17, 18].

Given the role of IL-6 in neuroinflammatory actions and its potential impact on cognitive function, it is not surprising that IL-6 secretion has been found to be altered in neurodegenerative dementia disorders linked to neuroinflammation. Examinations of post-mortem brain tissue from patients with Alzheimer’s disease (AD) have revealed activated glial cells as well as elevated expression of IL-6 adjacent to amyloid-β (Aβ)_1–42_ forming senile plaques [19, 20], a hallmark of AD pathology [21]. Increased numbers of activated microglia and elevated levels of IL-6 mRNA have also been found in the hippocampus of patients with dementia with Lewy bodies (DLB) [22], a neurodegenerative dementia disorder characterised by the presence of both senile plaques and α-synuclein occlusions [23]. Although studies investigating the impact of IL-6 on cognitive decline in AD and patients are few, a couple of studies support the idea. An association between increased levels of IL-6 and worse cognitive performance has been shown in patients with Parkinson’s disease (PD) and dementia [24] (a condition resembling DLB in regard to neuropathological changes and symptoms [23]) and patients with PD with cognitive impairment [18]. Patients with mild cognitive impairment (MCI), a condition associated with increased risk of dementia [25], show improvement of cognitive function as well as reduced peripheral IL-6 and tumour necrosis factor α (TNF-α) levels after repetitive physical exercise [26]. Additionally, increased levels of peripheral IL-1β and TNF-α, two glia-derived proinflammatory cytokines acting conjointly with IL-6 [27], have been found to be associated with marked cognitive decline in patients with AD [28, 29].

The altered brain expression of IL-6 in AD and DLB patients and the found link between elevated peripheral IL-6 and cognitive decline has led to studies attempting to monitor ongoing pathological neuroinflammation by analysing IL-6 concentration in CSF. These studies, in particular studies on patients with AD, have led to inconsistent results, with reports of increased levels [30–32], decreased levels [33] and unchanged levels [34, 35] of IL-6 in CSF.

To our knowledge, there is so far only one published study on CSF IL-6 levels in patients with DLB. That study showed a slight, although not significant, increase in CSF IL-6 levels in patients with DLB compared with age-matched controls [36]. Given this finding, together with the previous studies demonstrating increased neuroinflammation in relation to senile plaque as well as the many studies showing an impact of IL-6 on cognition, we find it likely that IL-6 plays a role in DLB pathology. To further investigate this hypothesis and to further explore whether DLB pathology affects CSF IL-6 levels differently compared with AD pathology, we here compared IL-6 CSF concentrations in patients with DLB, patients with AD and control subjects without dementia and examined the potential association between the IL-6 levels and biomarkers correlating with neuropathological changes characteristic of AD and DLB. We also examined the relationship between CSF IL-6 levels and Mini Mental State Examination (MMSE) scores to investigate whether IL-6 may be an underlying factor involved in the cognitive changes seen in patients with DLB.

## Methods

### Patients

The study included dementia patients and control subjects without dementia recruited through the Neurology Clinic and the Memory Clinic at Skåne University Hospital, Malmö, Sweden. The cohort has been described previously [37, 38]. It consisted of 36 control subjects without dementia, 45 patients with AD and 29 patients with DLB. The control subjects without dementia were individuals with mild cognitive symptoms, but these symptoms were not to the extent of a formal MCI or dementia diagnosis. Clinical diagnoses were made according to the American Psychiatric Association’s *Diagnostic and Statistical Manual of Mental Disorders, Fourth Edition* [39], combined with the diagnostic criteria of the National Institute of Neurological and Communicative Disorders and Stroke and the Alzheimer’s Disease and Related Disorders Association [40] for probable AD. Diagnoses of probable DLB were made according to the DLB consensus criteria [23, 41]. The cognitive status of the patients and the control subjects without dementia was evaluated using the MMSE [42]. The CSF AD biomarkers (Aβ_1–42_, total tau [T-tau], tau phosphorylated at threonine 181 [P-tau181]) were analysed according to clinical routine by commercial enzyme-linked immunosorbent assay (ELISA) kits (Innogenetics, Ghent, Belgium). Quantification of CSF α-synuclein levels was performed by the use of sandwich ELISA kits (Invitrogen, Carlsbad, CA, USA) as previously described [38, 43]. Individuals with acute or chronic inflammatory conditions (14 % of control subjects without dementia, 11 % of patients with AD and 13 % of patients with DLB) were excluded from the study. All individuals provided their informed consent, either (1) by use of a passive consent procedure whereby consent for retrospective use of banked clinical samples and data were assumed if individuals did not actively retract permission, as instructed in repeated local press advertisements; or (2) by active written informed consent. Informed consent was documented in two separate registries, including the patient’s medical chart, and in the local clinical research database. The study protocol was approved by the local ethics committee at Lund University and carried out in accordance with the Declaration of Helsinki.

### IL-6 quantification analysis

Levels of IL-6 in CSF were measured with a human proinflammatory 4-plex ultra-sensitive electrochemiluminescence immunoassay (Meso Scale Discovery, Rockville, MD, USA). This immunoassay is designed to analyse IL-6, interferon γ, TNF-α and IL-1β; however, only IL-6 levels were within detection range and are therefore the only values presented in this article. Standards and samples were analysed in duplicates. The detection limit for IL-6 was 0.055 pg/ml.

### Statistical analysis

Statistical analysis was performed using IBM SPSS software (version 20.0 for Windows; IBM, Armonk, NY, USA). The Kolmogorov–Smirnov test was used to assess the normality of distributions. The IL-6 levels were non-normally distributed within the three different groups and were therefore transformed into logarithms before analysis. One-way analysis of variance (ANOVA), followed by the Bonferroni post hoc correction (comparisons for n = 3), was used for comparisons between the three patients groups. Spearman correlation test were used for correlation between MMSE and IL-6 and the correlation between IL-6 and AD markers as well as α-synuclein were analysed using the Pearson correlation test. The results are presented as mean ± standard deviation. *p* < 0.05 was considered significant.

## Results

### Characteristics of individuals included in the study

The demographic data of individuals included in the study are presented in Table 1. The number of women and men was roughly the same in the control and DLB patient groups, but the AD patient group consisted of more women than men. The patients with dementia scored significantly lower than control subjects without dementia on the MMSE (*p* < 0.001). Both patients with AD and patients with DLB had significantly lower CSF levels of Aβ_1–42_ than control subjects (*p* < 0.001 and *p* < 0.001, respectively). P-tau and T-tau were significantly increased in patients with AD (*p* < 0.001 and *p* < 0.001, respectively), but not in patients with DLB, compared with control subjects without dementia. Levels of α-synuclein were significantly lower in CSF from patients with DLB than in control subjects without dementia. The patients with AD and patients with DLB were significantly older than control subjects without dementia (*p* < 0.001 and *p* < 0.001, respectively), but there were no age differences between the two patient groups.

**Table 1:** Characteristics of individuals included in the study

Variable	Control	AD	DLB
Number of subjects (M/F)^a^	36 (16/20)	45 (13/32)	29 (12/17)
Age (yr)^a^	62 (44–82)	76 (61–86)***	74 (63–84)***
MMSE (score)^a^	29 (26–30)^b^	20 (7–29)^b,^***	20 (7–27)***
P-tau (ng/L)^c^	46.33 ± 18.37^d^	105.25 ± 41.76^e,^***	57.96 ± 20.47^f^
T-tau (ng/L)^c^	287.68 ± 133.62^g^	823.10 ± 340.67^f,^***	412.69 ± 169.36^f^
Aβ_1–42_ (ng/L)^c^	713.45 ± 213.58^g^	352.71 ± 101.59^f,^***	457.31 ± 195.32^f,^***
α-Synuclein (pg/ml)^c^	693.61 ± 194.71	781.68 ± 222.25^b,^***	585.01 ± 216.44^b,^**
IL-6 (pg/ml)^c^	1.17 ± 1.10	1.37 ± 1.09	0.90 ± 1.23**

*Aβ*_*1–42*_ amyloid-β peptide 1–42, *AD* Alzheimer’s disease, *DLB* dementia with Lewy bodies, *IL* interleukin, *MMSE* Mini Mental State Examination, *P-tau* phosphorylated tau, *T-tau* total tau

^a^Data are presented as median (range)

^b^Data missing for one participant

^c^Data are presented as mean ± SD

^d^Data missing for 15 participants

^e^Data missing for 13 participants

^f^Data missing for three participants

^g^Data missing for 14 participants

***Indicates a significant difference at the *p* < 0.001 level compared with controls

**Indicates a significant difference at the *p* < 0.01 level compared with controls

### Cerebrospinal fluid levels of IL-6 are decreased in patients with DLB

**Fig. 1:**
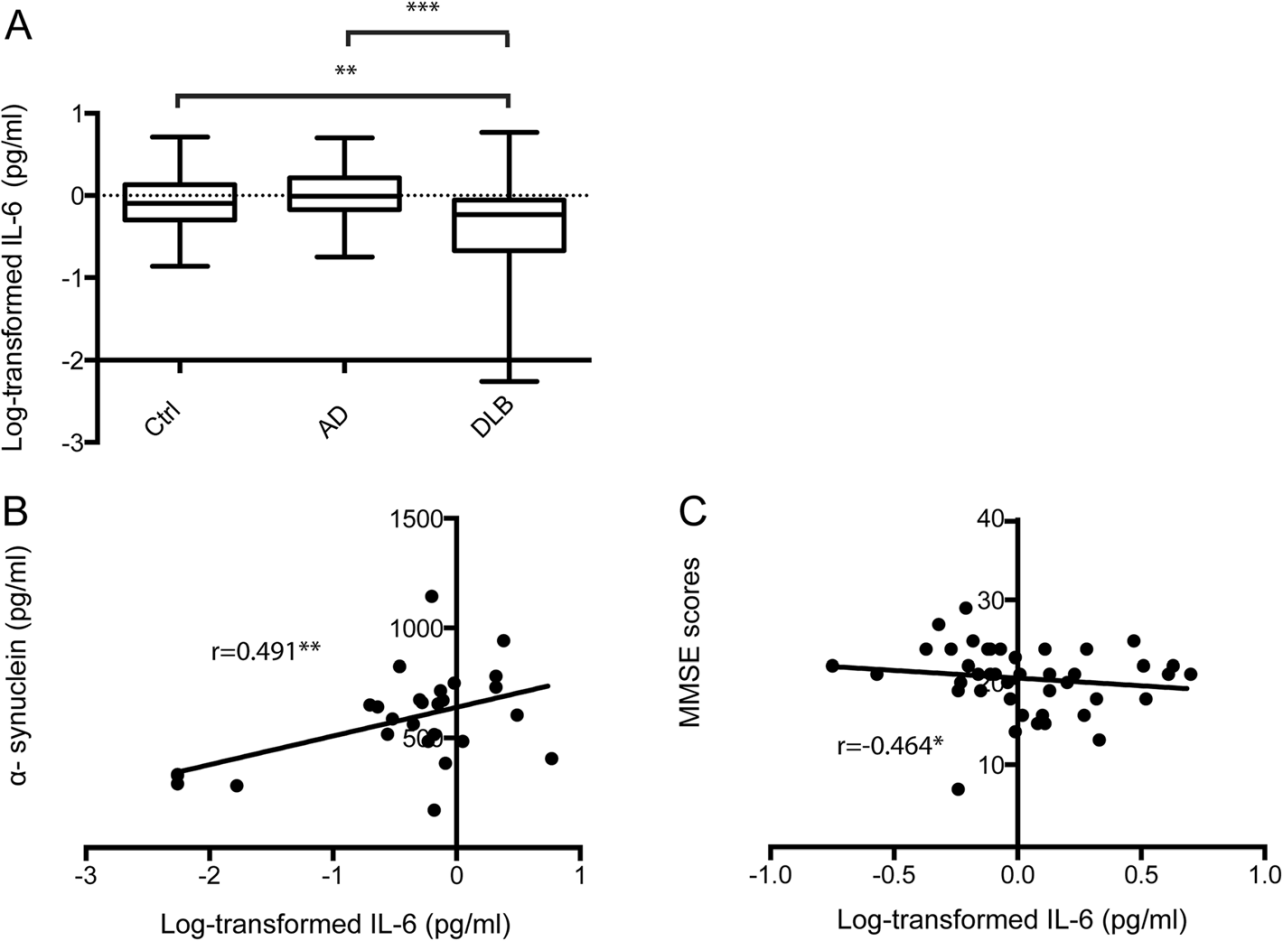
Log-transformed interleukin-6 (logIL-6) levels in cerebrospinal fluid (CSF) of control subjects without dementia (Ctrl), patients with Alzheimer’s disease (AD) and patients with dementia with Lewy bodies (DLB). **a** Box plot represents the mean ± standard deviation of logIL-6 levels in Ctrl, AD and DLB. **b** Plot shows correlations between CSF levels of α-synuclein and logIL-6 in patients with DLB (*p* < 0.01). **c** Plot demonstrates the negative correlation between Mini Mental State Examination (MMSE) score and logIL-6 in patients with DLB. *Significant difference at *p* < 0.05 level; **significant difference at *p* < 0.01 level; ***significant difference at *p* < 0.001 level

Our first aim was to determine whether IL-6 levels are altered in patients with DLB compared with patients with AD and control subjects without dementia. Because the IL-6 levels were not normally distributed, we transformed the IL-6 values by taking the logarithms (log) of the data before analysis. A multiple-comparisons analysis of log-transformed IL-6 values revealed significant differences between groups (*p* < 0.001 by ANOVA). Patients with DLB had significantly lower log-transformed IL-6 values than patients with AD and control subjects without dementia (*p* < 0.001 and *p* = 0.002, respectively), whereas the log-transformed IL-6 values of patients with AD did not differ from those of control subjects without dementia (Fig. 1a). The analysis further showed that gender had no impact on log-transformed IL-6 values in any of the analysed groups. Although we found no relationship between age and IL-6 levels, we considered the fact that control subjects without dementia were significantly younger than patients with DLB. Thus, to avoid potential biases, we analysed the log-transformed IL-6 values with age as a covariate (analysis of covariance [ANCOVA]). The analysis showed that the log-transformed IL-6 levels were still significantly lower in patients with DLB than in patients with AD and control subjects without dementia (*p* < 0.001 and *p* = 0.006, respectively). Finally, we analysed CSF from a randomly selected subset of individually age-matched patients with DLB and control pairs (n = 21). Values of log-transformed IL-6 in this subset of patients with DLB were also significantly lower than those of control subjects without dementia (*p* = 0.003).

### Cerebrospinal fluid levels of IL-6 correlate with MMSE and α-synuclein in patients with DLB

Next, we analysed the association between log-transformed IL-6 and biomarkers for AD and synucleinopathy. We found no correlation between log-transformed IL-6 and the AD markers Aβ_1–42_, P-tau and T-tau in control subjects without dementia, patients with AD or patients with DLB. We did, however, find a significant positive correlation between log-transformed IL-6 and α-synuclein in patients with DLB (*r* = 0.383, *p* = 0.048) (Fig. 1b), but not in patients with AD or control subjects without dementia. Finally, we investigated whether log-transformed IL-6 correlates with cognitive decline in patients with AD and patients with DLB. We detected a significant negative correlation between MMSE score and log-transformed IL-6 in patients with DLB (*r* = −0.464, *p* = 0.01) (Fig. 1c) and a trend to significance in patients with AD (*r* = −0.260, *p* = 0.089), but not in control subjects without dementia. Correlation analysis of the pairwise age-matched DLB patient and control subgroup showed that the IL-6 correlation with α-synuclein in patients with DLB stayed significant (*r* = 0.601, *p* = 0.006), whereas the significance between IL-6 and MMSE was lost and only a trend toward significance remained (*r* = −0.417, *p* = 0.060).

## Discussion

In line with the many previous preclinical and clinical studies demonstrating a link between cognition and IL-6, we show that CSF levels of IL-6 in patients with DLB significantly and negatively correlated with MMSE score. A similar, although not significant, association trend between these two variables was also seen in patients with AD. These findings indicate that increased IL-6 levels are linked to cognitive disabilities in patients with DLB and patients with AD and could be interpreted as further evidence of involvement of neuroinflammatory processes in both of these disorders. However, our results also show that patients with DLB displayed significantly lower CSF concentrations of IL-6 than both control subjects without dementia and patients with AD. This result inevitably contradicts the idea that neuroinflammatory processes, or at least IL-6 secretion, is a prominent feature of DLB pathology. Interestingly, a lack of protuberant neuroinflammatory processes in patients with DLB, as opposed to in patients with AD, has been described before. As noted in the Introduction section, studies imply that Aβ_1–42_ pathology underlies the altered IL-6 levels in patients with AD because elevated expression of IL-6 has been found in close vicinity to Aβ_1–42_ plaques in these patients [19, 20]. If this scenario holds true, one would expect that patients with DLB, who frequently also have Aβ_1–42_ plaques, would show elevated CSF concentration of IL-6. However, in a previous brain tissue study, researchers compared the amount of activated microglia in patients with AD and patients with DLB and found that, although activated microglia were present in all cortical regions of patients with AD, the number of activated microglia in patients with DLB did not differ from numbers found in control subjects without dementia [44]. The authors of the study pointed out that their patients with DLB, just like patients with DLB in general [23], did not display neurofibrillary tangles (NFT) characteristic of AD and suggested that NFT pathology rather than Aβ_1–42_ pathology is the underlying cause of the neuroinflammatory processes in AD [44]. This reasoning would explain the difference in IL-6 levels between patients with AD and patients with DLB in our study, as the patients with DLB showed unaltered P-tau concentrations (considered to be indicative of few or no NFT), whereas the P-tau levels in the patients with AD were significantly elevated (considered to be indicative of NFT presence).

Quantification of IL-6 in CSF from patients with DLB has (to our knowledge) been performed only once previously. That study, in contrast to ours, demonstrated a slight, non-significant increase in IL-6 levels [36]. The discrepancy between the previous finding and our results could be explained by the fact that the earlier study used a different IL-6 assay, but other variables may also be accounted for. For example the mean MMSE score of patients with DLB in the earlier study was lower than the mean MMSE score in our DLB patient groups (18.0 ± 4.8 vs 20.4 ± 5.5). Admittedly, the difference in MMSE is small, but in view of our findings demonstrating an increase in IL-6 levels in patients with lower MMSE scores, it may well be that an analysis of CSF from DLB patient groups with lower mean MMSE scores fails to reveal a potential disease-induced decrease in IL-6 levels. Further, the control subjects without dementia included in our study were significantly younger than the patients with DLB and patients with AD, which is a limitation, particularly considering that previous studies have demonstrated an age-dependent increase of IL-6 levels in CSF [45] and serum (for review, see [46]). However, no significant correlation between age and CSF IL-6 levels was found in our study, a finding also reported by other researchers [17, 24, 47]. The IL-6 levels were still significantly decreased in patients with DLB compared with both control subjects without dementia and patients with AD when the IL-6 values were analysed after age adjustment (ANCOVA). In addition, the levels of IL-6 in patients with DLB were significantly lower than those of patients with AD, who were within the same age range as the patients with DLB. Nevertheless, to exclude any potential cofounding effect of age, we selected 21 age-matched DLB patient–control pairs. Analysis of CSF from this subcohort still showed decreased levels of IL-6 in patients with DLB compared with control subjects without dementia (*p* = 0.003). From a clinical perspective it is important to point out that DLB has a male preponderance [48] and that our DLB cohort therefore should be considered atypical. However, because levels of IL-6 were unaffected by gender in all patient groups, we concluded that gender preponderance did not affect the results in this study.

Further, the size of our cohorts is rather small, which undoubtedly is a limitation of the study. Another limitation is the fact that we had access only to CSF from the individuals included in the study and therefore were not able to analyse IL-6 levels in serum or blood in the same individuals. Indeed, a comparison of IL-6 levels in blood and CSF could potentially indicate whether the IL-6 source is of peripheral or brain parenchymal nature. We would therefore like to emphasise the importance that future ante-mortem clinical studies on the implication of IL-6 in DLB and AD pathology should include larger cohorts as well as analysis of both CSF and serum and blood.

In recent years, levels of α-synuclein have been proposed as a biomarker for synucleinopathy, as patients with PD, DLB and multiple system atrophy often show decreased CSF levels of the peptide compared with patients with AD [1, 49–53] and controls [52, 54, 55]. It should be pointed out, however, that a couple of studies have shown contradicting results, with patients with DLB displaying unaltered [56, 57] or even increased [58] levels of α-synuclein compared with patients with AD. Additionally, in two studies researchers reported unaltered α-synuclein levels in patients with DLB compared with controls [49, 58], although the mean α-synuclein value in one study was lower than in controls. The α-synuclein values in patients with DLB included in our study were significantly decreased, which is in line with results in the majority of previous studies. Although the mean value of α-synuclein in patients with AD was increased, it did not reach significance. This discrepancy seen in our results and others’ could be due to several factors, including diagnostic criteria and cohort sample sizes. The fact that all the previously reported CSF α-synuclein studies used different analytical methods, including in-house ELISAs with various antibodies, mass spectrometry and Luminex technology (Luminex, Austin, TX, USA), is most probably also an important aspect to consider and highlights the importance of finding a global ‘gold standard’ when analysing CSF α-synuclein in the future. Nevertheless it should be underscored that the majority of studies, regardless of analytical method, point towards α-synuclein as a promising biomarker for distinguishing synucleinopathies such as DLB and PD with dementia from other forms of dementia.

Our study shows a positive correlation between IL-6 and α-synuclein levels in CSF from patients with DLB. This finding could be viewed from the perspective that lowered α-synuclein concentration is thought to mirror the synucleinopathy characteristic of entrapment of α-synuclein in the brain parenchyma. If this hypothesis holds true, IL-6 secretion must decrease as α-synuclein pathology increases. Such a scenario would be in line with our results showing decreased levels of IL-6 in patients with DLB compared with patients with AD and control subjects without dementia. The suggested scenario, however, inevitably also contradicts the earlier results demonstrating activated microglia and enhanced mRNA IL-6 levels adjacent to α-synuclein occlusions in the hippocampus of patients with DLB and patients with PD [22]. In vitro studies have additionally shown increased microglial activation [59, 60] and IL-6 secretion [59] in the presence of α-synuclein. Moreover, studies of patients with PD, another neurodegenerative disorder characterised by synucleinopathy, display microglial activation, increased mRNA expression of IL-6 [22, 61] and elevated CSF levels of IL-6 [30, 62]. With regard to these findings, and considering the fact that we found increased IL-6 levels in patients with DLB with very low MMSE scores, it is important not to rule out the possibility that IL-6 plays a vital role in DLB pathology. Future studies using animal and cell culture models are required, however, to fully understand the impact of IL-6 on brain cell integrity and function in relation to DLB pathology.

## Conclusions

Our study, by demonstrating lower CSF IL-6 levels as well as a correlation between IL-6 and α-synuclein in patients with DLB but not in patients with AD, underscores the differences in pathological and disease mechanisms between these two neurodegenerative dementia forms. Future studies with larger cohorts are required to ratify the potential use of IL-6 as a clinical biomarker to distinguish DLB pathology from AD pathology in early dementia stages.

The negative correlation we found between IL-6 and MMSE score in patients with DLB, as well as the tendency toward a correlation in patients with AD, adds to the growing evidence pointing towards a potential role of this cytokine in cognitive alterations.

## Abbreviations

Aβ_1–42_: amyloid-β peptide 1–42
AD: Alzheimer’s disease
ANCOVA: analysis of covariance
ANOVA: analysis of variance
CSF: cerebrospinal fluid
DLB: dementia with Lewy bodies
ELISA: enzyme-linked immunosorbent assay
IL: interleukin
logIL-6: log-transformed interleukin-6 values
MCI: mild cognitive impairment
MMSE: Mini Mental State Examination
NFT: neurofibrillary tangles
PD: Parkinson’s disease
P-tau: phosphorylated tau
TNF-α: tumour necrosis factor-alpha
T-tau: total tau
